# The Impact of GAGs, Cross-Link Maturity and Telopeptides on the Formation of a Porcine Collagen-Based Hydrogel

**DOI:** 10.3390/gels11090695

**Published:** 2025-09-01

**Authors:** Monika Šupová, Šárka Rýglová, Tomáš Suchý, Margit Žaloudková, Martin Braun

**Affiliations:** Department of Composites and Carbon Materials, Institute of Rock Structure and Mechanics, Czech Academy of Sciences, 182 09 Prague, Czech Republic; ryglova@irsm.cas.cz (Š.R.); suchy@irsm.cas.cz (T.S.); zaloudkova@irsm.cas.cz (M.Ž.); braun@irsm.cas.cz (M.B.)

**Keywords:** collagen, cross-linking, hydrogel, telopeptide, structure

## Abstract

Collagen hydrogels serve as biomimetic scaffolds that closely resemble the natural extracellular matrix, thus providing an ideal 3D biocompatible environment for cells. However, based on our previous experience, not all collagen isolates are capable of gelling, which appears to depend on the type, origin, species, age and sex of the source animal and the collagen isolation method applied. We therefore decided to evaluate porcine collagen-rich materials isolated from two different porcine genotypes applying two different specific isolation methods, and to analyse other main components, i.e., lipids and glycosaminoglycans, as well as amino acid composition and structural and morphological properties. While all the collagen isolates obtained were subjected to the gelling process, only one of them successfully gelled. In addition, the gelling ability of this isolate was confirmed repeatedly on collagens that were isolated from other pigs of the same porcine genotype. The results revealed that the gelling process proceeds via cooperation between the composition and the structure of the collagen isolate. With respect to the composition, one of the most important factors in terms of the success of the gelation process of collagen isolates concerns elevated glycosaminoglycan contents. The structural factors that characterise collagen isolates, i.e., cross-links (immature and mature) and their mutual ratio, as well as the presence of telopeptides, strongly impact the progress of the gelling process and the resulting character of the hydrogel structure. All these factors are influenced by the isolation procedure.

## 1. Introduction

Hydrogels comprise polymer-based highly swollen network structures that have the ability to encapsulate cells and bioactive molecules and to efficiently transfer oxygen, nutrients, and metabolites. Various degradable natural and synthetic hydrogels serve as scaffold materials [1,2] due to their ability to mimic the natural environment. Their properties, including the chemical and physical structure, cross-linking density, diffusivity and porosity, allow for their tailored tissue-specific design via the exploitation of their adjustable, controllable swelling properties, degradation rates and, consequently, their mechanical properties. Natural hydrogels [3] such as fibrin gel [4], collagen (COL) [5], COL/chondroitin sulphate gel [6], gelatine (GEL) and alginate [7], etc., have received considerable attention due to their ability to provide instructive biological cues, in contrast to synthetic materials. The formation of hydrogels and their properties, including their viscosity and texture, are determined primarily by the structure, molecular size, temperature and pH of the system [8]. COL is used widely in the cosmetic, pharmaceutical and food industries due to its favourable water-bearing properties and ability to form hydrogels. COL hydrogels provide an in vivo 3D environment that is suitable for the study of cell–matrix interactions during the formation of proto-tissue. The networks of cell-seeded collagen hydrogels are remodelled by the cells and provide a directional template that can be degraded and replaced by a cell-synthesised extracellular matrix [9]. The formation of COL hydrogels, as well as their mechanical and degradation properties, are influenced and controlled by a wide range of factors [10].

COL hydrogels are often prepared from cold acid-solubilised monomers (tropocollagen) via neutralisation and warming aimed at inducing physical cross-linking [10]. The gelation of COL proceeds due to fibrillogenesis—a self-assembly process that commences with the self-association of the triple helix [11]. The end-to-end and side-to-side assembly of monomers results in the formation of fibrils, which further aggregate into fibril bundles, fibres and a highly ordered structure. Collagen fibrillogenesis is strongly influenced by the nature of the COL monomers, as well as a host of physical factors [9]. Telopeptides (the non-helical parts of the collagen molecule) play a crucial positive regulatory role in the reconstitution of fibrils. Yang and Kaufman [12,13] found that 1.0 mg/mL pepsin-solubilised collagen took 10–15 min to reach its equilibrium storage modulus for gelation at 37 °C, whereas concerning acid-solubilised collagen (containing telopeptides), gelation occurred within 5 min at 37 °C. Prockop and Fertala [14] revealed that the de novo assembly of collagen I monomers into fibrils depends on the interactions of specific binding sites in the telopeptide and helical regions of monomers. Telopeptides, which are rich in hydrophobic residues, are helpful in terms of inter-micro fibrillar interaction. The intra- and inter-molecular cross-links in acid soluble collagen (ASC) are evidently richer than those in pepsin soluble collagen (PSC) [15].

Fibrillogenesis is induced via the neutralisation of the pH and the increasing of the temperature, while increases in the ionic strength lead to a decrease in the fibrillogenesis rate. The pH and ionic strength impact the net surface charges of COL molecules. A low pH results in repulsive electrostatic interactions between the COL molecules, thus causing them to dissolve homogeneously [16]. Neutral pH conditions lead to the weakening of the electrostatic repulsion via the neutralisation of cationic and the dissociation of anionic functional groups. The kinetics of fibril self-assembly are further influenced by changes in the electrostatic, hydrophobic and covalent interactions between the monomers, thus resulting in a range of fibril sizes [9]. These interactions are strongly influenced by the concentrations of both the COL and the solvent [15]. The changes in the interactions impact the morphologies of the fibrils, including the fibril diameter and length, and ultimately affect the microstructure and properties of the collagen gel [17,18]. The fibril diameter obtained from changes in the pH ranges from 80 to 220 nm. The fastest rates of fibrillogenesis occur between pH of 6.9 and 9.2, with no significant changes in the fibril diameter [19]. Joseph [20] synthesised natural collagen hydrogels in differing chemical environments within biologically relevant pH (5.5–8.5) and temperature (25 °C and 37 °C) ranges, and investigated how these variables affected the structure and morphology of the collagen fibrils. Increasing the pH resulted in thinner collagen fibrils at lower polymerisation temperatures and thicker collagen fibrils at higher polymerisation temperatures. Furusawa et al. [21] demonstrated that temperature is the main factor that influenced the gelation kinetics of a COL solution. The kinetics of COL fibrillogenesis proceed in two stages: the nucleation process and the subsequent growth of the nuclei and the aggregation of the COL fibrils that results in the 3D network structure of the hydrogel [22]. The temperature also affects the water-mediated hydrogen bonding between the collagen molecules [23]. Holmes et al. [24] revealed that fibrils that formed at lower temperatures (20 °C) evince larger diameters than those that formed at higher temperatures (34 °C). Two procedures can be distinguished according to the initial conditions: the so-called “warm start” (warming and then neutralisation), which provides fewer unbonded filaments than the “neutral start” procedure (neutralisation and then warming).

The gel formation of COL and the gel network structure differ significantly from those of GEL. The COL gelation process is characterised by a lag phase during which the primary aggregates of the COL molecules are nucleated. Microfibrillar aggregation subsequently commences with the lateral aggregation of subunits that are induced by changes in the ionic strength, the pH and the temperature up to 37 °C up to the attainment of equilibrium. In contrast, the basic mechanism of GEL is related to the reverse coil-to-helix transition induced via the cooling of solutions to below 30 °C, during which the helices formed are similar to the COL triple helix, but without attaining equilibrium. The gelation processes for both COL and GEL are thermo-reversible, but in opposite directions: COL gels melt via the lowering of the temperature, whereas GEL gels melt via the raising of the temperature [25]. Shu et al. [26] investigated the impacts of amino acid residues in four collagen-like peptides on the kinetics of the triple helix conformation, self-assembly into higher order structures and the maintenance of the gel network structure. Collagen-like peptides of different sequences were designed to elucidate the role of polar, non-polar and ionisable residues.

Based on our previous experience, not all collagen isolates are capable of gelling [27], a process that appears to depend on various factors, including the type, origin, animal species, age and sex of the source animal and the collagen isolation method applied. The gelling ability, the progress of the gelation process, and the resulting properties of the hydrogel are, therefore, particularly important, especially with concern to tissue engineering studies in which collagen scaffolds are often used as matrices aimed at optimising and guiding cell growth. A number of studies have already addressed this issue. A study by Holder et al. [28] confirmed that the manipulation of the gelation conditions involved in sol–gel transition provides for a unique degree of control over the rheological properties of the rat collagen gel network. Moraes et al. [29] investigated the effect of temperature and pH on the technological properties of bovine collagen hydrolysates over time and the enhanced gelling ability and water-bearing capacity of the hydrogels that formed from these hydrolysates. Kim and Bonassar [30] demonstrated that the gelation pH exerts profound impacts on the cellular activity, the rat collagen fibril structure and the mechanical properties of collagen-based gels over both the short and long terms. These studies focused on evaluating the physical properties so as to assess the transition point between viscoelastic liquid and viscoelastic solid behaviour, which occurs following the establishment of a sample spanning network at the gel point, and the resulting mechanical properties. This study focuses on porcine collagens isolated from skin. Porcine collagen is almost identical to human collagen but is less likely to provoke allergic reactions [31]. This gelling isolate was used in one of our previous studies to prepare inks for 3D bioprinting purposes [32,33]. Bioprinting using collagen hydrogel bioinks holds significant potential in terms of the imitation of tissues and the production of artificial organs in regenerative medicine.

This study evaluated porcine collagens isolated from two different porcine genotypes applying two different specific isolation procedures. The yield of both isolation procedures was collagen-rich materials containing additional components such as glycosaminoglycans and lipids. The extraction of pure collagen was beyond the scope of this study. The aim of this research was to investigate how the composition of collagen-rich isolates (amino acids, lipids, glycosaminoglycans) and their structural properties (the polypeptide patterns of the analysed materials, secondary structure of the collagen) affect the ability to form hydrogels. To the best of our knowledge, no other such comprehensive studies using porcine collagen have been conducted to date.

## 2. Results and Discussion

### 2.1. Preparation of the Collagen-Based Hydrogels

**Figure 1:**
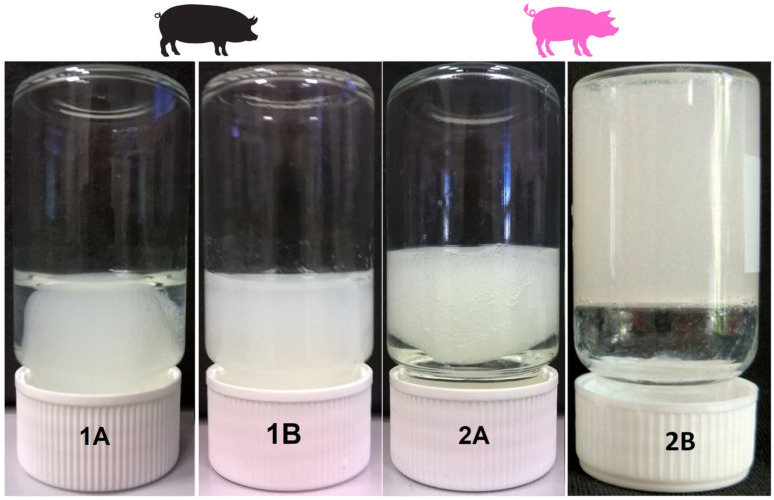
Formation of hydrogels from two different porcine sources (1 and 2) isolated by means of two different procedures (A and B). Detailed descriptions of the porcine origin, genotype and isolation procedures applied for the isolation of the source collagen are provided in Section 4.1.

A comparison of the gelling abilities of the various collagen-rich isolates (1A, 1B, 2A and 2B) is shown in Figure 1. Only collagen isolate 2B was able to form a 3D hydrogel structure. The hydrogels of the other isolates (1A, 1B and 2A) exhibited incomplete or unstable gelation; their resulting “hydrogel” masses were formed within a period of up to 4 h, whereas hydrogel 2B was formed within up to 30 min. The 1A, 1B and 2A “hydrogels” formed a shape-unstable “hydrogel-like” structure. The 1A and 2A “hydrogels” were unable to retain all the water in the hydrogel structure, while “hydrogel” 1B failed to form a compact gel structure and remained in the form of a suspension. Therefore, it was not possible to characterise the conventional physical properties of these materials (1A, 1B and 2A), e.g., the rheological behaviour, gel strength, swelling or thermal stability. This study therefore focused on the analysis of the compositions and structures of the isolated collagens and the determination of the factors that influence the successful formation of a 3D hydrogel structure.

The gelling ability of collagen isolate 2B was verified by isolating collagens from four other different pig individuals (2B-2, 2B-3, 2B-4 and 2B-5) of the same pig genotype. All of these isolates gelled as successfully as did 2B.

### 2.2. Characterisation of the Porcine Collagen-Rich Lyophilisates

#### 2.2.1. Compositions of the Amino Acid and GAGs

The amino acid and centesimal compositions of the lyophilised collagen-rich isolates (1A, 1B, 2A and 2B) are presented in Table 1. The compositions of the successfully gelled 2B(1–5) collagens are presented in Table 2. Typical collagen lyophilisates can contain not only protein (collagen and traces of other proteins) but also residual lipids and glycosaminoglycans (GAGs) in various quantities [34], which, given the isolation procedures used, is also evident in our isolates. The high data scatter of lipids and GAGs is thought to be related to local inhomogeneities. The total protein content, as represented by amino acids (AAs), varied within the range 55–71 wt%. The distribution of AAs was defined as the number of specific AA residues in 1000 AA units.

The results of the contents of the various amino acids, as listed in Table 1 and Table 2, did not allow for the identification of a demonstrable trend that would serve to explain the influence of either the individual amino acids or their cumulative sums based on the type (basic, acidic, polar, non-polar) or their mutual ratios (non-polar/polar and acidic/basic) upon successful gelation. However, the lipid and protein contents (Table 2) and their variability did not exhibit any trends that could serve to explain the gelling ability.

**Figure 2:**
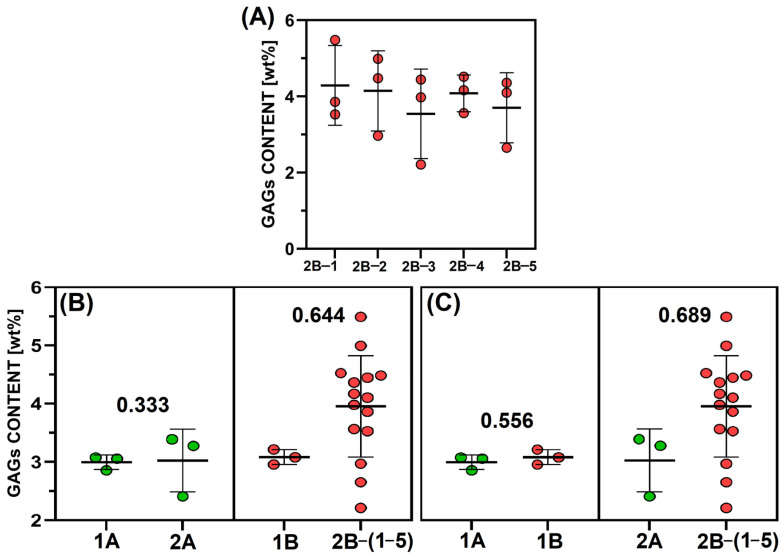
Comparison of the GAGs content in the lyophilised collagen: (**A**) isolated via procedure B from five individuals of pig genotype 2 (*n* = 3); (**B**) evaluation of the effect of the pig genotype (1 versus 2) (*n* = 3 or 15 (pooled data from A); (**C**) evaluation of the impacts of the isolation procedures (A versus B) (*n* = 3 or 15 (pooled data form A)). The effect size is indicated by the rank-biserial correlation coefficients (**B**,**C**). No statistical significance was determined (the Dunn nonparametric test, **A**; Mann–Whitney test, **B**,**C**).

The situation differed with concern to the GAGs content (Figure 2). Since the content of GAGs did not change upon the application of the isolation procedure (B) to the pig genotype (2) (Figure 2A), the data was merged for further statistical analysis purposes. The influence of the pig genotype (1 and 2) on the content of GAGs in the various isolates is evident from Figure 2B. The GAGs content exhibited no significant difference (small effect size) following the application of isolation procedure A (left), whereas the application of isolation procedure B (right) led to a significant difference in the GAGs content (large effect size). The influence of the isolation procedures (A and B) on the GAGs content of the isolates is clear from Figure 2C. Whereas concerning porcine genotype 1 (left), the GAGs content was of medium quantity, after considering the percentage difference, the difference was interpreted as minimal. The large effect size of Porcine genotype 2 (right) was interpreted as indicating a significant difference.

#### 2.2.2. Structural Characterisation

**Figure 3:**
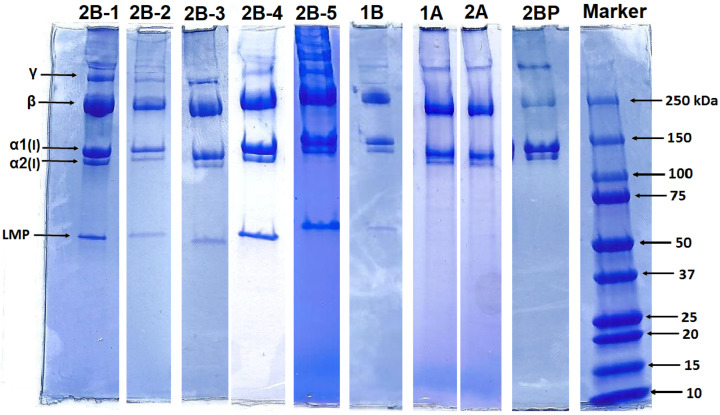
Polypeptide patterns of the analysed materials as determined via the SDS-PAGE method.

Figure 3 shows the electrophoretic profiles of all the COL materials studied as obtained by means of polyacrylamide gel electrophoresis (SDS-PAGE), a technique that is used to separate molecules, primarily proteins, based on their size and charge. Molecules migrate under an electric field through a medium of polyacrylamide gel. The protein patterns evinced α1 and α2 chains with average molecular weights in the range 130–110 kDa; their dimer (β chain) forms are visible at ~250 kDa and trimer (γ chain) forms are visible above 300 kDa. The band belonging to the β-component was revealed to comprise the major component of the SDS-PAGE profile. All the COL materials exhibited identical electrophoretic profiles that are typical of collagen type I. The profiles of the B-line materials, except for 2BP (1B and 2B1–5), also included low-molecular-weight peptide (LMP) components visible at ~50–55 kDa. These low molecular weight components were most likely attributable to the α-unit fragment of the collagen [35]. In addition to LMPs, peptide fragments of non-collagenous protein residues may also have been present in the COL isolate. As can be seen from Figure 3, SDS-PAGE did not allow for the distinction of significant quantitative differences between the isolates.

**Figure 4:**
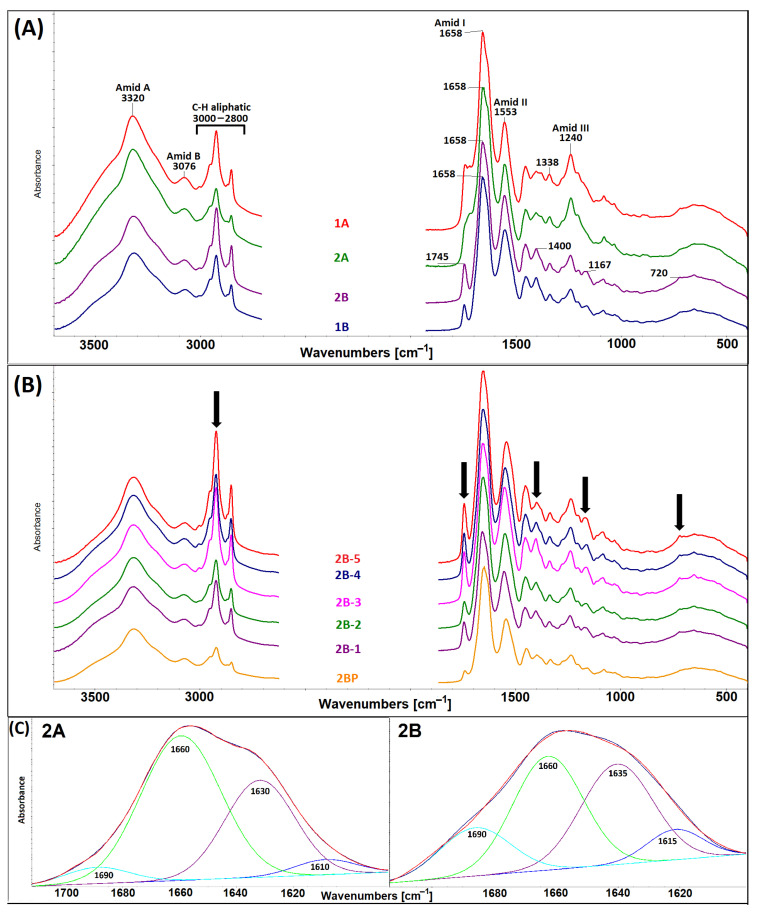
(**A**) Comparison of the infrared spectra of the collagens isolated from two different porcine genotypes (1 and 2) and isolated via two different procedures (A and B); (**B**) Comparison of the infrared spectra of the collagen 2B series isolated from five different pig individuals and isolated using pepsin (2BP); (**C**) Examples of the amide I deconvolution of the ATR-FTIR spectra of the collagen lyophilisates obtained from the same porcine genotype (2) and isolated by means of the two different procedures (A and B).

A further method used for structural analysis purposes comprises infrared spectrometry (FTIR), which can be used as an analytical technique for the description of the collagen secondary structure and for the interpretation of changes in the collagen secondary structure following the application of various processes (isolation, cross-linking, denaturation, sterilisation, etc.). The FTIR spectra of collagens 1A, 1B, 2A and 2B and those of collagens 2B-(1–5) including 2BP are shown in Figure 4A and Figure 4B, respectively. The FTIR spectra of the collagen lyophilisates shown in Figure 4A contain 5 amide bands typical of proteins [36,37]. Amide A, related to N-H stretching, is centred at 3320 cm^−1^, amide B, visible at ~3076 cm^−1^, is attributed to the stretching vibrations of the N-H bonds in the secondary amides. The amide I (~1658 cm^−1^) and amide II (~1553 cm^−1^) bands originate from combinations of C=O stretching vibrations with N-H bending vibrations and N-H bending vibrations coupled with C-N stretching vibrations, respectively. Moreover, the existence of a triple helical collagen structure was demonstrated by the presence of a quartet of bands at ~1205, 1240 and 1280 cm^−1^ (amide III) and 1338 cm^−1^ [34,38]. The FTIR spectra in Figure 4B reflected the fact that both the acid-soluble collagens (2B1–5) and the pepsin-soluble collagen (2BP) exhibit a triple helical conformation character. Nevertheless, a number of subtle differences were evident in the peak intensities or positions due to certain differences in the inter- and intra-molecular interactions that were evident between the two types of collagen. The bands visible in Figure 4B and marked with black arrows (spectral regions 2800–3000, 1745, 1455, 1065 and 720 cm^−1^) are related to the presence of lipids. These bands varied in intensity in the spectra of the collagens isolated by means of procedure B (Figure 4B). The amide I region of the collagen spectrum can be deconvoluted into several distinct bands [37]. The deconvolution of the amide I region revealed four bands, i.e., at ~1690, 1660, 1630–1635 and 1610–1615 cm^−1^. As can be seen in Figure 4C, the character of the amide I band differed with respect to procedures A and B.

**Figure 5:**
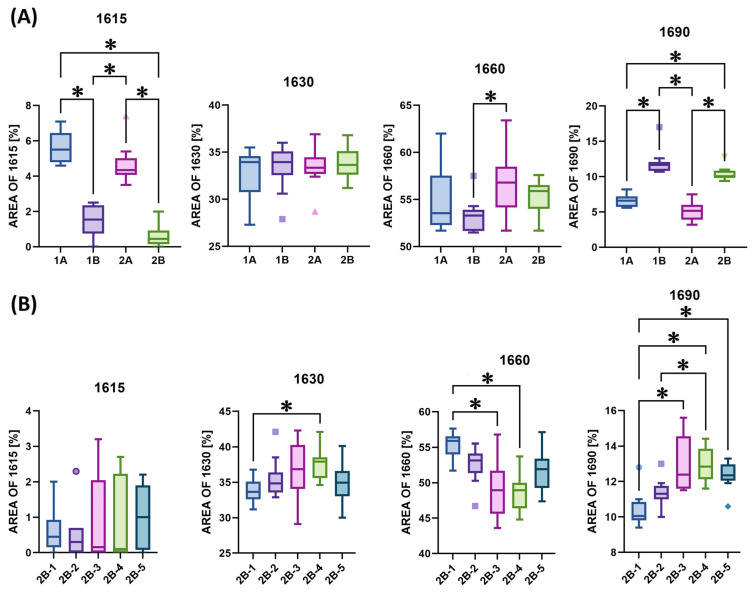
Box plots (*n* = 20) that compare areas 1615, 1630, 1660 and 1690 following the deconvolution of amide I: (**A**) isolates 1A, 1B, 2A and 2B; (**B**) isolates 2B-1, 2B-2, 2B-3, 2B-4 and 2B-5. The pairs with *p*-values of <0.05 (the Dunn nonparametric test with correction) are marked with “**∗**”.

**Figure 6:**
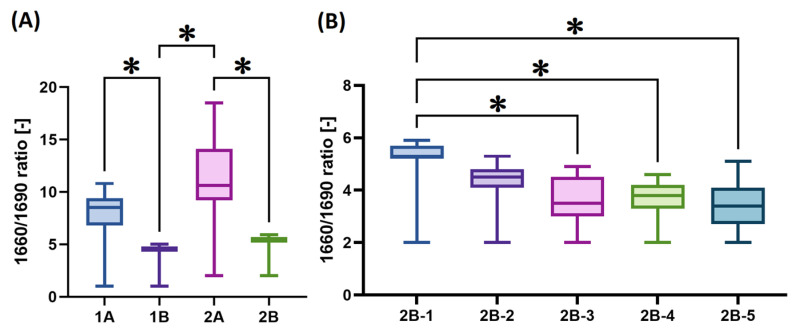
Box plots (*n* = 20) that compare the 1660/1690 area ratio following the deconvolution of amide I: (**A**) isolates 1A, 1B, 2A and 2B; (**B**) isolates 2B-(1–5). The pairs with *p*-values of <0.05 (the Dunn nonparametric test with correction) are marked with “**∗**”.

Comparisons of the spectral components obtained via the deconvolution of the amide I band, expressed as a percentage of the total area of amide I, of all the materials studied are shown in Figure 5. The band at 1690 cm^−1^ is associated with the β-turn and antiparallel β-sheet structure [37]. The band at ~1660 cm^−1^ provides the strongest evidence for a triple helix. In contrast, band ~1630 cm^−1^ was assigned to the left-handed 3–10 helix in the denatured state. The spectral component at ~1615 cm^−1^ can be assigned to aromatic rings in the aromatic AAs, which may be more spectroscopically active in gelatin, such as the disintegrated collagen state [39]. Of the various underlying bands that make up the amide I spectral peaks, two are of particular interest in terms of the study of collagen maturity: 1660 and 1690 cm^−1^. Collagen maturity is calculated as the area ratio of the 1660/1690 sub-bands, which are assigned to mature and immature collagen cross-links, respectively, and are naturally present in collagen [40,41]. This characteristic refers to the parameters of the source animal (species, sex, age). The comparison and statistical evaluation of the 1660/1690 area ratio are shown in Figure 6.

Statistically significant differences were observed primarily for bands 1615 and 1690 (Figure 5A). The collagens isolated via procedure A exhibited more significant percentage differences with concern to the medians between the individual structural states (i.e., 1615 versus 1630 versus 1660 versus 1690); see Figure 4C and Figure 5A. In addition, these collagens also exhibited a higher degree of data scatter, which was related to the lower homogeneity of these matrices. The B procedure exhibits less of the 1615 component and more of the 1690 component, which is further reflected in the 1660/1690 ratio in Figure 6A; thus, isolation applying the B procedure yielded less mature collagen than via the application of procedure A. In the case of the B series (Figure 6B), statistically significant lower ratios were evident for materials 2B-3, 2B-4 and 2B-5. The 2B series collagens isolated from five different pigs (1–5) evinced statistically significant differences in the 1630, 1660 and especially 1690 spectral components (Figure 5B) and in the 1660/1690 ratio (Figure 6B), but only within the range of values shown in Figure 5A and Figure 6A.

#### 2.2.3. Morphological Assessment of the Internal Hydrogel Structure

**Figure 7:**
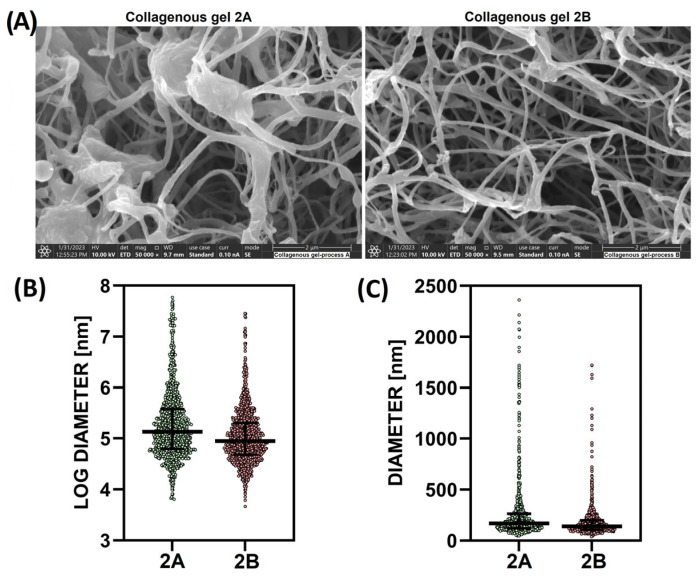
(**A**) Representative SEM images of the two collagen gels—source: porcine collagen of genotype 2 isolated via the two different procedures (A and B); mag. 50,000×, bar 2 μm (top), (**B**) Fibre diameter of the collagen isolated via procedures A and B following logarithmic transformation, (**C**) Statistically significant differences were determined between the transformed means of the two samples (*t*-test; *p* < 0.0001, *n* = 1188).

Aimed at forming an understanding of the influence of the isolation procedure on the resulting internal structure of the hydrogels, detailed morphological analyses were performed using SEM on hydrogels prepared from porcine genotype 2 collagen isolated using the two different methods—A and B (Figure 7A). Many of the irregular structures (nodules and fibres) in the 2A collagen gel were observed to be unevenly distributed, whereas the fibres in the structure of collagen gel 2B were more homogeneous in terms of the fibre diameter (Figure 7B). The fibre diameters in both hydrogels were statistically significantly different. The fibre diameter in material 2A (193.3 ± 1.0 nm) was statistically significantly higher than in material 2B (153 ± 1.0 nm); see Figure 7C. Due to its size, the internal structure that formed in the 2A gel appeared to have a lower active surface area upon which to bind water molecules, thus resulting in a lower retention capacity; see Figure 1. The internal structure of the 2B gel was observed to be more isotropic and homogeneous, with a high water retention capacity and overall shape stability. However, the final mechanical properties, porosity and potential cell viability were affected by variations in the pH as shown in our previous studies [28,29].

### 2.3. Factors for Successful Gelation

**Figure 10:**
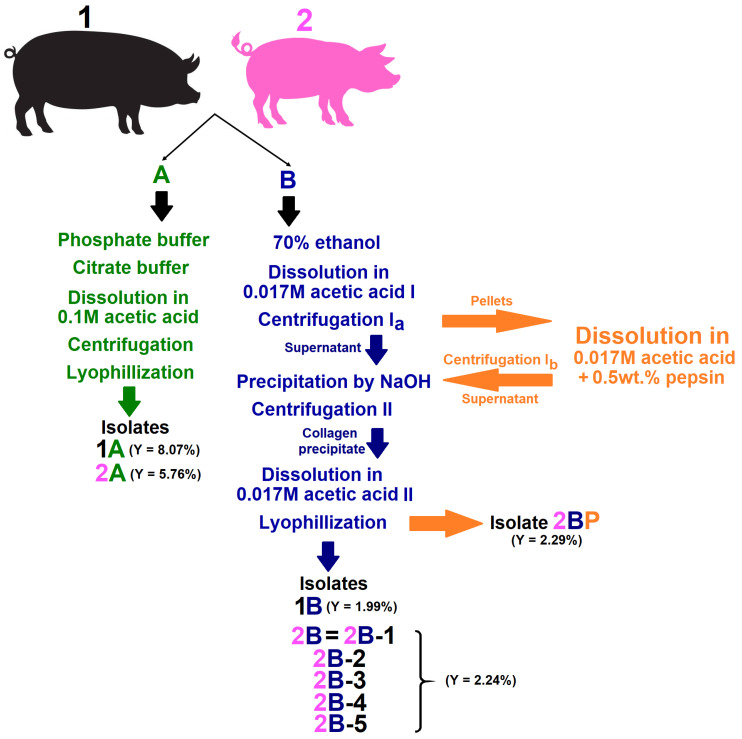
Scheme of the isolation of the porcine collagens with the yield (Y) *w*/*w* of dry collagen to wet skin.

Extraction and isolation act to transform or alter the original COL to a greater or lesser extent [42]. However, following the pre-extraction process (e.g., using a phosphate buffer or 70% ethanol; see Figure 10), isolated collagen may still contain small amounts of GAGs, non-collagenous proteins and lipids. Collagen also contains variable amounts of telopeptides (non-helical parts), mainly in the form of monomers with varying amounts of cross-linked components, dimers, trimers and certain higher order components [43].

**Figure 8:**
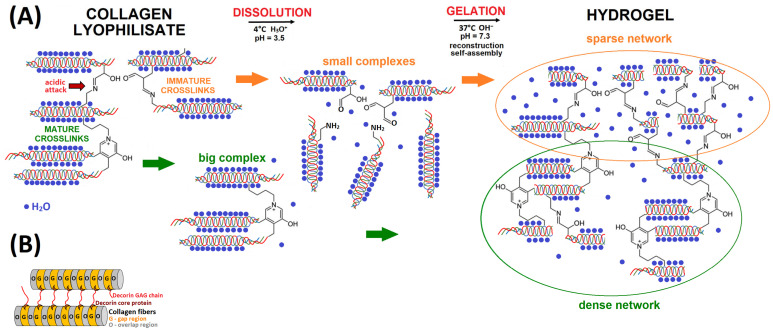
Scheme of the formation of collagen hydrogels depending on the type of cross-links (**A**) and GAGs that bind between nearby collagen fibrils (**B**).

Cross-links in collagen occur both within and between microfibres. Interestingly, the super-twisted nature of collagen microfibrils is maintained by the non-helical telopeptide regions. The location of divalent, immature cross-links occurs between the telopeptide lysines and hydroxylysines in the end-overlap domain of adjacent molecules (intrafibrillary), where aldimine-containing cross-links (Schiff bases) are formed [44]; see Figure 8A. The location of the pyridinoline moiety is crucial in terms of its role in the biomechanical properties of tissues since it must link more than two collagen molecules if it is to provide additional strength to the collagen matrix [44]. Mature cross-links can be trivalent or tetravalent [40] and are formed interfibrillarly (Figure 8A). Mature cross-links are formed enzymatically from immature cross-links via reactions with other amino acids, particularly histidine, so as to form pyrrole and pyridinium cross-links [9].

The pig skin collagens isolated in our study applied two differing processes referred to as “Procedure A”, following a study by Pešáková et al. [45] and “Procedure B”, as described by Bell et al. [46] and as mentioned in Section 4.1 and illustrated in Figure 10. The advantages of these two processes comprise their simplicity and the utilisation of low-cost, environmentally and cell friendly, laboratory safe agents. Both the A and B processes are based on extraction in acetic acid but at different concentrations and with different cross-link stages. In addition, the two processes differ in terms of the pre- and post-extraction step protocols. With concern to the isolation of the collagen, several methods, including extraction with neutral salt, acid and enzymatic solutions [47] relating to various animal sources [48] were described and reviewed. The collagen extraction process uses more often dilute acid solubilisation, typically acetic acid, to break the weaker hydrogen bonds between the collagen molecules [49]. Dilute acid has the potential to destroy the intermolecular bonds and Schiff bases, thus increasing the repulsive charges on the triple helix so as to swell the collagen fibres. Collagen extracted in acidic solutions retains the triple helix with preserved telopeptides. All bivalent immature cross-links are both acid and heat labile [40]. Davison et al. [50] concluded that the treatment of collagen with 0.05 M acetic acid for 1 h or less leads to the profound rearrangement of the intermolecular bonds in both soluble and insoluble collagens. These processes are strongly influenced by the origin of the acids (organic/inorganic), the concentration, temperature and time [51]. Dissolution at low pH and low temperatures results in the cleavage of immature divalent cross-links and the subsequent release of these individual components into the acidic solution (Figure 8A). The cleavage of aldimine-containing cross-links with acids produces smaller complexes containing aldehydes and amines. Mature cross-links are not attacked by acids and therefore remain in solution as spatially distinct complexes.

Gelation is induced by changing the pH to 7.3 and increasing the temperature to 37 °C. The formation of hydrogels is thus a process that involves in vitro fibril reconstruction and the reconstitution of collagen molecules in the 3D space (Figure 8A). Interstitial water, which is directly bound to the collagen helix even in the lyophilised state, comprises an integral part of the collagen molecule [52]. COL self-assembly, its alignment and long-range order depend on the molecular size of the collagen helices, as well as on the preservation of the non-helical end regions (telopeptides) [43]. Spatially distinct components that contain preserved mature cross-links exert a significantly enhanced influence on the final arrangement of collagen fibrils during the gelation process and the resulting homogeneity of the gel. The homogeneity of the gel affects its shape stability and mechanical properties. A high degree of collagen cross-linking exerts a negative impact on water retention in the hydrogel since cross-linking significantly reduces the ability of collagen to bind water molecules [53].

Procedure “A” involved the application of phosphate and citrate buffers to remove the non-collagenous proteins and acid-labile components that contained immature cross-links, respectively. The subsequent application of acetic acid resulted in the isolation of the collagen with a higher (more concentrated) proportion of mature cross-links, irrespective of the pig genotype (1 or 2); see Figure 6A. This is consistent with a higher degree of hydroxylation (see Table 1). This resulted in the formation of a gel that was not dimensionally stable and did not have sufficient water retention properties; see 1A and 2A in Figure 1. Paradoxically, collagen clusters that contain mature cross-links surrounded by immature cross-links can also be isolated using procedure “B”, although the concentration of acetic acid applied in this method is lower than that in procedure “A”.

It can be concluded from the results that the type of cross-links (immature and mature) exerts a much greater influence on the formation, resulting character and properties of the inner structure of hydrogels than might at first appear. A sparse network of immature cross-links appears to be more favourable for the formation of the optimal 3D gel structure. The formation of the internal structure of hydrogels depending on the type of cross-links is shown in Figure 8A. The variability of the internal structure (dense/sparse) correlates with the SEM observation (Figure 7), where the character of the internal structure is influenced by the isolation procedure applied. However, the hydrogel prepared from isolate 1B formed a homogeneous mass with sufficient water retention but insufficient shape stability (Figure 1). This indicated that isolates 2B-(1–5) encompassed an additional factor that allowed for the preparation of an optimal hydrogel matrix.

The gelation of COL solutions is a complex process that potentially involves other macromolecules [11]. Some authors have ‘enriched’ purified collagens by adding GAGs so as to enhance gel formation; the addition of GAGs serves to improve the mechanical strength [54], stiffness [55] and porosity, which also promotes cell proliferation [45,56]. In addition, GAGs promote water retention, bind growth factors and cytokines, and contribute to the gel properties of the extracellular matrix [57]. GAGs comprise negatively charged mucopolysaccharide compounds composed of repeating disaccharide units attached to a polypeptide core so as to link two collagen fibrils/fibres; they provide the intermolecular force in the collagen-GAG matrix [58]; see Figure 8B, and are able to attract water molecules. As can be seen from the results shown in Table 1 and Table 2 and the processing of the statistical data (Figure 2), lyophilised collagen 2B contained a higher concentration of GAGs than the other isolates (1A, 1B and 2A). The higher concentration of GAGs in collagen isolate 2B compared to isolate 1B may have comprised the additional factor (referred to above) in the explanation of the formation of the resulting 3D hydrogel structure with optimal water retention and shape stability. The same trend was demonstrated by Pešáková et al. [45] whose insoluble collagen (isolated via procedure A) failed to form a gel in contrast to their acid-soluble collagen (isolated via procedure B) which did so. Insoluble collagen contains terminal peptides that, in turn, contain intermolecular cross-links. The authors suggest that the kinetics of lattice gel formation and retraction depend on the amount of collagen cross-links and the concentration of proteoglycan in the culture medium.

**Figure 9:**
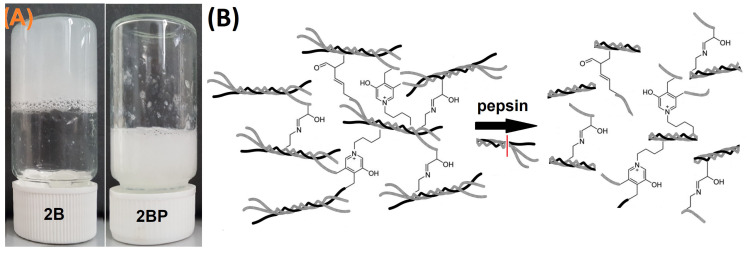
(**A**) Formation of a hydrogel from lyophilised collagen 2B isolated via standard procedure B using pepsin (2BP); (**B**) a scheme of the formation of a collagen hydrogel with and without telopeptides.

The important role of telopeptides in intrafibrillar and interfibrillar cross-linking was discussed earlier in this text. Aimed at verifying this premise, an experiment was performed in which collagen isolate 2B was exposed to pepsin (2BP) during isolation; see Figure 10. The FTIR spectrum (Figure 4B) and SDS-PAGE polypeptide pattern (Figure 3) did not reveal any significant differences between isolate 2BP and the other 2B isolates. As can be seen from Figure 9A, isolates without exposure to pepsin are able to form gels, but not following its application. In the case of 2BP, no compact mass was formed, and the material behaved as a suspension. A schematic representation of the role of the telopeptides in the formation of the 3D hydrogel structure is shown in Figure 9B. The collagen extracted in the acid solution retained the triple helix with telopeptides. The fact that pepsin cleaves the telopeptide parts of the collagen molecule, which affects the bond system (both immature and mature), and consequently disrupts the chemical formation of fibrillar cross-links and the cohesion of collagen molecules comprises an important requirement for the formation of 3D hydrogel systems. It has been proved by several researchers that acid soluble collagen following the removal of telopeptides exhibited both a relatively lower fibre formation rate [59,60] and a weaker collagen gel strength [61].

The limitation of this study lies in the biological variability, which significantly impacted the collagen cross-links and composition, thus affecting the mechanical properties and stability. Extraction from animal sources often results in low batch-to-batch consistency [35]. Factors including age, tissue type and a range of genetic variations influence the types and amounts of collagen cross-links formed and the composition and, ultimately, determine the strength, flexibility and resistance to degradation of the tissue. Collagen cross-linking increases with age, particularly during development and maturation [62], which leads to stiffer and more resistant collagen, but may also act to reduce the tissue elasticity and increase susceptibility to fractures. Different tissues feature varying types of collagen and cross-linking patterns. For example, tendons have a higher proportion of mature cross-links than skin, which contributes to the tensile strength of the former [40]. Such variations in collagen genes and the related enzymes potentially impact the efficiency of collagen synthesis and cross-linking [63]. This may then lead to differences in the properties of the collagen between individuals, even between those with the same tissue type. In addition, various diseases act to disrupt collagen cross-linking. For example, diabetes often leads to the excessive non-enzymatic glycation of collagen, which alters its properties and contributes to tissue damage [64]. Factors such as diet and lifestyle also influence collagen cross-linking; for example, nutrient (e.g., vitamin C) deficiencies may impair the cross-linking of collagen [65].

## 3. Conclusions

The results of this study suggest that not all porcine collagen-rich isolate is capable of gelling or forming a gel with sufficient structural properties. Our study performed on two types of porcine skin and two isolation procedures demonstrated that the ability to gel and the progression of the gelling process depend on both the genotype of the pig and the extraction procedure employed.

The isolation procedure selected is an important influencing factor since it potentially impacts the final ratio of immature to mature cross-links in the collagenous isolate. The hydrogel prepared from the collagen isolate containing a higher proportion of immature cross-links formed a homogeneous mass with sufficient water retention and shape stability. This study confirmed the positive role of telopeptide moieties in collagen molecules in terms of the successful formation of a hydrogel structure. Our study further demonstrated the fundamental impact of GAGs—the collagen lyophilisate that contained the highest concentration of these mucopolysaccharides exhibited the highest degree of gelling ability. No correlation was detected with the contents or characters (basic, acidic, polar or non-polar) of the various amino acids, nor with the concentration of lipids.

Therefore, the gelling process appears to be influenced by the interaction of various parameters of the content and structural characteristics of collagen isolates. The isolation procedure, which affects the ratio of mature to immature cross-links, as well as the presence of telopeptides in the collagen isolate, appear to be particularly important factors. The glycosaminoglycan content also contributes positively to gelation. The potential for adding GAGs to collagen hydrogels and determining their optimal concentrations provides almost unlimited possibilities with regard to future research in this area.

However, the assertion that these conclusions automatically apply to collagens isolated from any animal species (e.g., mammals, fish, etc.) or from any mammalian species (e.g., cow, rat, etc.) is not entirely accurate and requires confirmation via the conducting of further studies. It is important to recognise that collagen isolates can naturally contain, in addition to collagen itself, other components. The presence and concentration of these components depend on the isolation procedure used and can affect the gelation process. The isolation procedure used, as well as the source of the skin, influences the structural properties of the collagen itself and also the concentration of these components.

## 4. Materials and Methods

### 4.1. Isolation of Collagen

The skin collagen-rich isolates were extracted from two different pig genotypes, i.e., the two most commonly bred genotypes in the Czech Republic. The porcine skin of both genotypes was obtained from a local slaughterhouse (Steinhauser s.r.o, Skalice nad Svitavou, Czech Republic), both were from controlled breeding and aged 6 months. Full thickness skin was obtained from the back and pre-treated via scalding so as to remove the hair. The first porcine genotype comprised the Přeštice Black Pied pig referred to as “1” and the second was the Czech Improved White pig referred to as “2”. Collagen isolates were obtained via the application of two different procedures referred to as “A” according to the literature [45] and “B” as described by other authors [46]. Procedure “A” yields insoluble collagen solubilised under denaturation conditions (40 °C) while procedure “B” yields acid soluble collagen. The gelling ability of collagen isolate 2B was verified by means of isolating collagens from four other different pig individuals (2B-2, 2B-3, 2B-4 and 2B-5) of the same pig genotype. In order to verify the role of the telopeptides in the formation of the hydrogel structure, collagen was isolated from porcine genotype 2 using procedure B under exposure to pepsin, referred to as “2BP”. Isolates 1A, 1B, 2A and 2B were obtained by processing skins from three pig individuals (*n* = 3), thus representing mixed samples, while isolates 2B-2, 2B-3, 2B-4, 2B-5 and 2BP were obtained from individual pigs (*n* = 1). The isolation procedures are shown schematically in Figure 10.

#### 4.1.1. Procedure A

Cut and cleaned pieces of skin were stirred in a phosphate buffer (1 g skin/10 mL, pH 7.4, applied 3 × 24 h, room temperature) so as to remove fat, non-collagenous proteins and residual saccharides. This was followed by citrate buffer (1 g/10 mL, pH 3.7, applied 3 × for 24 h, at 4 °C) treatment aimed at removing the collagen fraction that was soluble in this buffer. Following pre-treatment, the skin was washed three times with water, skin/water ratio of 1:10 (*v*/*v*), then briefly crushed in a blender, stirred in a 0.1 M acetic acid solution (1 g/20 mL, 24 h, at 4 °C) and centrifuged (18,000× *g*/40 min). Finally, the collected supernatant was frozen at −25 °C and lyophilised.

#### 4.1.2. Procedure B

The cut and cleaned pieces of skin were stirred in 70% ethanol (*v*/*v*) solution (1 g skin/10 mL, 30 min), washed 3 times with water and, subsequently, in an acetic acid solution 1:1000 (*v*/*v*) (1 g/20 mL, 48 h). Following centrifugation I_a_ (18,000× *g*/55 min), the collagen in the collected supernatant was precipitated with 0.1 M NaOH solution at 6:1 (*v*/*v*) to a neutral pH = 7 (monitored by a pH electrode) and re-centrifuged (centrifugation II) for 45 min. The resulting collagen precipitates were then dissolved in 1:1000 (*v*/*v*) acetic acid solution, frozen at −25 °C and lyophilised. All the collagen lyophilisates were stored in a freezer at −25 °C.

#### 4.1.3. Procedure B with Exposure to Pepsin

One gram of skin residues (pellets) following the first centrifugation I_a_ were stirred in 20 mL of acetic acid/pepsin solution (0.5 g of pepsin in 100 mL of 0.017 M acetic acid) for 24 h at 20 °C. The supernatant was neutralised with 0.1 M NaOH solution at 6:1 (*v*/*v*) immediately following centrifugation I_b_ (to halt the enzymatic activity of pepsin, monitored by means of a pH electrode); isolation then continued as in procedure B; see Figure 10.

### 4.2. Preparation of the Collagen Hydrogels

Lyophilised COL I was dissolved in 0.02 M acetic acid at a concentration of 5 mg/mL and stored at 4 °C for 5 days. The COL suspension was homogenised using a disintegrator in order to break down the aggregates and to enhance homogeneity (10,000 rpm, 2 min), accompanied by the monitoring of the solution temperature. The hydrogel was prepared by mixing the COL suspension with the medium at a ratio of 3:2 (*v*/*v*) ratio. The medium contained Dulbecco’s Modified Eagle Medium, foetal bovine serum solution (10 wt.%—stock concentration) and sodium bicarbonate solution (7.5 wt.%—stock concentration) mixed at a ratio of 1:1:0.05 (*v*/*v*/*v*). All the chemicals were supplied by Sigma-Aldrich Merc corp., Burlington, MA, USA. The collagen suspension with the medium was allowed to polymerise for at least 30 min at 37 °C in a normal atmosphere so as to attain a pH of around 7.3 via the buffering action of bicarbonate; the pH of ~7.3 was an expected value based on previous studies. The final concentration of COL in the gels was 3 mg/mL.

### 4.3. Characterisation of the Porcine Collagen-Rich Lyophilisates

Amino acid analysis was performed using an Ingos AAA 400 analyser; the collagenous materials were hydrolysed in 6 M hydrochloric acid at 110 °C/23 h then evaporated and diluted in a solution (acetic acid, sodium chloride, thiodiglycol and sodium azide; pH = 2.2). The sulphur-containing amino acids were oxidised with peroxoformic acid at 5 °C for 16 h. The oxidation of methionine and cysteine converted them to methionine sulphone and cysteic acid, which survived the subsequent acid hydrolysis (Standard ČSN 46 7092-25, Annex No. 9 of Decree 124/2001 [66]). In place of ether-petroleum ether, dichloromethane, which is both heavier and immiscible with water was used; subsequently, a phase interface rapidly formed. This modification is suitable for determining fat in various animal materials (i.e., not only for dairy products). The hydroxyproline (Hyp) content was determined according to the ISO 3496:1994(E) standard [67] for meat and meat products. The hydrolysate was oxidised with chloramine B, followed by a quantitative reaction with p-dimethyldiaminobenzaldehyde; the Hyp content was detected spectrophotometrically at 558 nm (*n* = 5) using a Unicam UV 530 (Thermo Fisher Scientific, Waltham, MA, USA. Quantification was performed using a calibration curve; trans-4-Hydroxy-L-proline (56250-5G, Sigma-Aldrich, Merc corp., USA) was used as the standard. The GAGs content was quantified by means of high-performance liquid chromatography (HPLC) based on the formation of fluorescent N-acetylated hexosamine derivatives via reaction with a specific derivatising agent—OPA/3MPA solution. The derivatisation solution contained 250 μL of OPA solution (10 mg of o-phthaldialdehyde dissolved in 1 mL of methanol, HPLC grade), 82 μL of 3MPA (3-mercaptopropionic acid), 100 μL of NaOH (sodium hydroxide) and 2068 μL of 0.4 M borate buffer pH 10.2 (Agilent Technologies Inc., Santa Clara, CA, USA, cat. no. 5061–3339) to a final volume of 2.5 mL. A volume of 35 μL of the derivatisation agent was mixed intensively with 5 μL of each of the samples (following acid hydrolysis with 6 M hydrochloric acid and sodium borohydride reduction) for 1 min using a Vortex shaker (IKA) prior to injection into the Shimadzu LC10 ADvp HPLC system equipped with a C18 reversed-phase Luna Omega 5 μm Polar C18 100A LC 150 × 4.6 mm (Phenomenex Inc., Torrance, CA, USA) separation column with a compatible small guard column obtained from the same company. Mobile phase A consisted of 0.05 M sodium phosphate buffer (monobasic/dibasic), pH 7.2 in 25% methanol; mobile phase B consisted of methanol, water and tetrahydrofuran at a 70:30:3 volume ratio. A flow rate of 1.0 mL/min was applied during the gradient RP-HPLC run. The fluorescent derivatives of glucosaminitol and galactosaminitol were detected using a Shimadzu RF-10A XL fluorescence detector with the excitation/emission wavelength set at 337/454 nm. The lipid content was assessed via the Schmidt-Bondzyński-Ratzlaff method according to the Czech EN ISO 1735:2004 technical standard (https://www.iso.org/standard/35250.html (accessed on 28 August 2025)). The SDS-PAGE was performed using the Mini-Protean Tetra Cell electrophoresis system from BIO-RAD on TGX Miniprotean Precast Gel, 4–15% (BIO-RAD, Hercules, CA, USA), Coomassie Brilliant Blue-R was used as the dye for the detection of the bands of the individual components, separation voltage of 200 V was applied for 30 min; the collagen isolate was dissolved in acetic acid solution (0.1 M). FTIR with an attenuated total reflection (ATR) mode was used to evaluate the secondary structure and changes in the isolated collagen lyophilisates (iS50, Nicolet Instrument, Madison, WI, USA, diamond crystal). All the spectra were recorded in the middle spectral range of 4000–400 cm^−^^1^ with a resolution of 4 cm^−^^1^ via 64 scans). The number of analysed locations in the collagen lyophilisate was 10, aimed at verifying the homogeneity of the collagen lyophilisates. The spectra were evaluated and processed using OMNIC version 9 software with the Gaussian function for the amide I band deconvolution procedure. The initial calculation parameters for the curve fitting process were set via a combination of the secondary derivative method and Fourier self-deconvolution (standard deconvolution tools in OMNIC). The areas of the amide I bands were statistically evaluated. In order to visualise the morphology of the two collagen gels (2A and 2B), they were examined “in situ” (the preserved internal hydrogel structure due to fixation) via scanning electron microscopy (SEM) on a STEM Apreo S2 microscope (Thermo Scientific, Waltham, MA, USA) in high vacuum mode, standard application. An Everhart-Thornley detector was used in secondary electron mode at 10 keV with 50,000× magnification. The collagenous hydrogels were fixed with a fixative solution (the composition and preparation of this solution are described in [33]) for 2 h at room temperature followed by overnight fixation at 4 °C, further processed via multiple washing in a phosphate buffer and graded ethanol and acetone dehydration series in a Leica EM TP tissue processor (Specion s.r.o., Prague, Czech Republic), and dried in a Leica EM CPD300 (Specion s.r.o., Prague, Czech Republic) critical point dryer. The dried specimens were mounted on stubs using carbon adhesive stickers and sputter coated with Pt in an Ar atmosphere in the Leica EM ACE600 (Specion s.r.o., Prague, Czech Republic) coating system. In order to verify the homogeneity of the internal structures of the two hydrogels, 66 different points were scanned. Using 2 different 9-point grids (fixed intervals), 18 fibre diameter values were obtained from each image using ImageJ software (https://imagej.net/ij/ accessed on 30 July 2024). All the values obtained were statistically processed; see Section 4.4.

### 4.4. Statistical Analysis

Standard descriptive statistics and statistical procedures were employed to process the experimental data. Due to the risk of non-normal distribution, with concern to the larger datasets, the medians were calculated with the interquartile range so as to highlight the variability, whereas the mean and standard deviation (SD) were calculated for the small sample sizes (*n* = 3, GAGs content). Since it is not possible to test normality with such small samples, the mean was considered to be a more consistent and comparable value than the median. The data was presented using either box plots or scatter plots with the mean and SD shown. The normality of the data was verified primarily by means of the Shapiro–Wilk test and by constructing Q-Q plots. Homoscedasticity was tested applying the Levene test and the Bartlett test (for normally distributed data). This part of the statistical analysis was performed in Statgraphics Centurion XVII (StatPoint, Warrenton, VA, USA). A non-parametric analysis was applied since either the assumption of normality or homoscedasticity was violated. The Kruskal–Wallis test was performed with a subsequent post hoc test based on the Dunn test (correction for multiple comparison). The Mann–Whitney test was used for the two-sample (unpaired) comparisons. Statistical significance was accepted at *p* ≤ 0.05. This part of the statistical analysis was performed in GraphPad Prism software (ver. 10.3.1, GraphPad Software, San Diego, CA, USA). The effect sizes were calculated as rank-biserial correlation coefficients, which can be interpreted in the same way as the Pearson’s correlation coefficient, i.e., small effect (0.1–0.3), medium effect (0.3–0.5) and large effect (0.5–1.0). This part of the statistical analysis was performed in JASP (open-source, ver. 0.17.2.1, JASP Team 2023, University of Amsterdam, Amsterdam, The Netherlands). The sample size was not based on a power analysis, rather it was constrained by the availability of the biological samples, which determined the number of replicates feasible for each analysis.

Logarithmic transformation was applied to the fibre diameter data due to the skewed distributions. This is a simple statistical method that allows for the data distribution to be transformed from non-normal to normal [68]. The means and confidence intervals (95%) were calculated from the logarithmic data and back-transformed, i.e., the mean diameter was estimated as the geometric mean. The Student *t*-test was used for the two-sample comparison of the transformed data (assuming equal variances). *p* values of less than 0.05 were considered statistically significant.

## Figures and Tables

**Table 1:** Compositions of the COL lyophilisates isolated from two different pig genotypes (1 and 2) isolated via two different procedures (A and B).

Analysed Component	Numbers of AA Residues/1000 Units			
	1A	1B	2A	2B = 2B-1
* Aspartic acid (Asp) + Asparagine (Asn)	50	49	49	48
* Glutamic acid (Glu) + Glutamine (Gln)	77	78	76	77
Threonine (Thr)	16	18	16	18
Serine (Ser)	30	31	30	33
Glycine (Gly)	319	315	328	322
Alanine (Ala)	108	110	109	112
Valine (Val)	31	31	28	28
Isoleucine (Ile)	13	12	11	11
Leucine (Leu)	28	31	26	28
Cysteine (Cys)	2	3	2	2
Methionine (Met)	6	6	6	9
Tyrosine (Tyr)	4	5	4	3
Phenylalanine (Phe)	15	16	14	15
Lysine (Lys)	31	34	30	31
Histidine (His)	15	15	14	14
Arginine (Arg)	51	50	49	49
Proline (Pro)	118	121	122	123
Hydroxyproline (Hyp)	85	75	87	75
Degree of hydroxylation (%) *^a^*	42	38	42	38
Imidoacids *^b^*	203	196	209	198
Nonpolar AAs *^c^*	638	632	644	648
Polar AAs *^d^*	361	358	357	350
Acidic AAs (COO-) *^e^*	127	127	125	125
Basic AAs (=NH^+^, -NH^2+^) *^f^*	97	99	93	94
Ratio acidic AAs/basic AAs	1.31	1.28	1.34	1.33
Ratio nonpolar AAs/polar AAs	1.77	1.77	1.80	1.85
GAGs (wt%)	3.00 ± 0.12	3.08 ± 0.13	3.03 ± 0.54	4.29 ± 1.05
Lipids (wt%)	21.49 ± 1.75	26.65 ± 0.97	6.24 ± 0.18	47.83 ± 15.55
Protein (wt%)	55.93	70.40	69.40	54.26

* Asn and Gln are listed together with Asp and Glu since Asn and Gln were converted into Asp and Glu during hydrolysis. *^a^* Degree of hydroxylation = Hyp/(Hyp + Pro) × 100. *^b^* Imidoacids = Hyp + Pro. *^c^* Nonpolar = Ala + Val + Leu + Ile + Pro + Phe + Met + Gly. *^d^* Polar = Asp + Glu + Asn + Gln + Ser + Thr + Cys + Tyr + Hyp + His + Lys + Arg. *^e^* Acidic = Asp + Glu + Asn + Gln. *^f^* Basic = His + Lys + Arg.

**Table 2:** Compositions of the COL lyophilisate 2B series isolated from five different pigs of the same pig genotype (2B-1, 2B-2, 2B-3, 2B-4 and 2B-5).

Analysed Component	Numbers of AA Residues/1000 Units				
	2B-1	2B-2	2B-3	2B-4	2B-5
Asp + Asn	48	48	48	50	50
Glu + Gln	77	77	76	84	84
Thr	18	16	17	18	18
Ser	33	35	33	33	31
Gly	322	311	316	304	312
Ala	112	109	109	110	112
Val	28	25	26	24	24
Ile	11	12	12	11	11
Leu	28	29	28	29	29
Cys	2	2	2	3	3
Met	9	6	7	7	6
Tyr	3	8	7	5	3
Phe	15	13	15	16	16
Lys	31	31	29	30	30
His	14	13	12	13	14
Arg	49	46	49	49	48
Pro	123	137	120	129	127
Hyp	75	82	91	85	81
Degree of hydroxylation (%)	38	37	43	40	39
Imidoacids (Pro + Hyp)	198	219	211	214	208
Nonpolar AAs	648	636	633	630	637
Polar AAs	350	358	364	370	362
Acidic AAs (COO-)	125	125	124	134	134
Basic AAs (=NH^+^, -NH^2+^)	94	90	90	92	92
Ratio acidic AAs/basic AAs	1.33	1.39	1.38	1.46	1.46
Ratio nonpolar AAs/polar AAs	1.85	1.78	1.74	1.70	1.76
GAGs (wt%)	4.29 ± 1.05	3.92 ± 0.91	3.20 ± 0.62	3.35 ± 0.94	3.70 ± 0.92
Lipids (wt%)	47.83 ± 15.55	28.81 ± 1.78	27.47 ± 1.35	23.16 ± 3.03	20.25 ± 2.60
Protein (wt%)	54.26	62.95	61.92	67.89	76.68

## Data Availability

Data is contained within the article.
